# Image quality improvement in low‐dose chest CT with deep learning image reconstruction

**DOI:** 10.1002/acm2.13796

**Published:** 2022-10-09

**Authors:** Qian Tian, Xinyu Li, Jianying Li, Yannan Cheng, Xinyi Niu, Shumeng Zhu, Wenting Xu, Jianxin Guo

**Affiliations:** ^1^ Department of Radiology The First Affiliated Hospital of Xi'an Jiaotong University Xi'an Shaanxi P. R. China; ^2^ GE Healthcare, Computed Tomography Research Center Beijing P. R. China

**Keywords:** adaptive statistical iterative reconstruction V, deep learning image reconstruction, image noises, low‐dose chest CT

## Abstract

**Objectives:**

To investigate the clinical utility of deep learning image reconstruction (DLIR) for improving image quality in low‐dose chest CT in comparison with 40% adaptive statistical iterative reconstruction‐Veo (ASiR‐V40%) algorithm.

**Methods:**

This retrospective study included 86 patients who underwent low‐dose CT for lung cancer screening. Images were reconstructed with ASiR‐V40% and DLIR at low (DLIR‐L), medium (DLIR‐M), and high (DLIR‐H) levels. CT value and standard deviation of lung tissue, erector spinae muscles, aorta, and fat were measured and compared across the four reconstructions. Subjective image quality was evaluated by two blind readers from three aspects: image noise, artifact, and visualization of small structures.

**Results:**

The effective dose was 1.03 ± 0.36 mSv. There was no significant difference in CT values of erector spinae muscles and aorta, whereas the maximum difference for lung tissue and fat was less than 5 HU among the four reconstructions. Compared with ASiR‐V40%, the DLIR‐L, DLIR‐M, and DLIR‐H reconstructions reduced the noise in aorta by 11.44%, 33.03%, and 56.1%, respectively, and had significantly higher subjective quality scores in image artifacts (all *p* < 0.001). ASiR‐V40%, DLIR‐L, and DLIR‐M had equivalent score in visualizing small structures (all *p* > 0.05), whereas DLIR‐H had slightly lower score.

**Conclusions:**

Compared with ASiR‐V40%, DLIR significantly reduces image noise in low‐dose chest CT. DLIR strength is important and should be adjusted for different diagnostic needs in clinical application.

## 1 INTRODUCTION

Lung cancer remains the leading cause of cancer‐related death and has the lowest 5‐year survival rate of all cancer types.^1^ The US Preventive Services Task Force recommends annual screening for lung cancer with low‐dose CT (LDCT) in adults aged 50–80 years who have a 20 pack‐year smoking history and currently smoke or have quit within the past 15 years.^2^ The National Lung Screening Trial, the largest randomized clinical trial, reported a 20% reduction in the relative risk of lung cancer, as well as a reduction in all‐cause mortality from lung cancer with three annual LDCTs compared with three annual chest radiograms.^3^

Image quality is affected by radiation dose. Although increasing radiation dose may improve diagnostic ability, radiation dose itself may also increase cancer risk. Therefore, balancing image quality and radiation dose remains a challenge in LDCT lung cancer screening.

Despite refinements in CT hardware technology, increased image noise with traditional reconstruction methods, such as filtered back projection (FBP), has limited further dose reduction. As a consequence, several iterative reconstruction (IR) methods have been developed by various vendors, such as iDose and IMR by Philips, IRIS, SAFIRE, and ADMIRE by Siemens, AIDR‐3D, and FIRST by Canon as well as ASiR, ASiR‐V by GE.^4^, ^5^, ^6^ Typically, the stronger the IR level, the more “blotchy” the image looks. A pixilated and blotchy image appearance that has repetitively been described at high reconstruction levels constitutes a limitation of IR techniques and affects the evaluation of CT images and arguably also the interpretation of imaging findings.^7^ New reconstruction methods that could maintain image quality at reduced radiation dose are desirable. Recently, a deep learning image reconstruction (DLIR) algorithm (TrueFidelity, GE Healthcare) has been introduced. In this technique, deep convolutional neural network‐based models are used under low‐dose conditions to emulate standard‐dose FBP image texture. while providing low image noise, high spatial resolution, streak artifact suppression, and high contrast for easier detection of low contrast lesions (Revolution CT user manual, GE Healthcare). A commercially available DLIR uses a large set of standard‐dose FBP images of both phantoms and patients that are essentially artifact‐free with special corrections to train the software.^8^ In addition, low, medium, and high reconstruction strengths can be selected according to different dose and noise reduction requirements. However, it is yet to be established whether DLIR brings advantages when it applies to low‐dose chest CT.

The purpose of our study was to investigate the effects of DLIR with different reconstruction strengths on chest CT images in comparison with commonly used ASiR‐V40% in low‐dose chest CT.

## 2 MATERIALS AND METHODS

This was a retrospective study approved by the local institutional review committee. Written informed consent was obtained from all patients for using the images. Images were collected from all patients undergoing low‐dose chest CT for lung cancer screening examinations from May to August 2018. Cases with severe respiratory motion artifacts were excluded. Finally, a total of 86 patients were included in the study.

### 2.1 CT image acquisition

All patients were scanned on a 256‐slice CT scanner (Revolution CT, GE Healthcare, USA). Patients were scanned in supine position with headfirst and arms raised. During the scan, patients inhaled deeply and held breath in accordance with the machine instruction. The scanning parameters were as follows: tube voltage, 120 kVp; tube current range, 10–130 mA; noise index, 16; pitch, 0.992:1; rotation time, 0.5 s; reconstruction field of view, 36 cm; scan field of view, 50 cm with Large Body bowtie filter, reconstruction kernel: standard, non‐HD; and reconstruction thickness: 1.25 mm; ASiR‐V strength: 40%.

The datasets were reconstructed with the standard ASiR‐V40% and DLIR at low (DLIR‐L), medium (DLIR‐M), and high (DLIR‐H) strength levels with 1.25 mm axial section thickness. All reconstructed images were transmitted to an advanced workstation (AW4.7) for data measurement and image analysis.

### 2.2 Objective image analysis

Objective image quality analysis was performed on an AW4.7 workstation by a radiologist with 15 years of experience in CT imaging. The standard deviations (SDs) were defined as the objective image noise to reflect image quality. The CT attenuation values in Hounsfield unit (HU) and SDs of descending aorta, subcutaneous fat, erector spinae muscles, and lung tissue were measured centered on a plane 2 cm below the tracheal bifurcation. The region‐of‐interest (ROI) was placed on three consecutive image slices (centered, one slice above and below the centered slice) to reduce the measurement error, and the average value was taken as the final result. All objective image analyses were performed on axial images at the same plane of different reconstructed images using an ROI of 5 mm diameter avoiding artifacts and lung texture when measuring lung tissues. Lung tissue was measured under the pulmonary window (window width: 1500 HU; window level: −600 HU), and the rest were measured under the mediastinal window (window width: 350 HU; window level: 40 HU).

The volumetric CT dose index (CTDIvol) and dose length product (DLP) values were recorded after the CT scans. The effective radiation dose received by patients was calculated using the product of DLP and effective dose conversion factor *K* (*K* = 0.014 mSv/mGy cm for chest CT).^9^

### 2.3 Subjective image analysis

All images were also subjectively evaluated by two qualified radiologists (with 5 and 10 years of experience in CT imaging) on a 5‐point scale in terms of noise, artifacts, and visualization of small structures. Detailed grading rules are listed in Table 1. The radiologists were blinded to the patient information and image reconstruction techniques. The visualization of small structures, including the display of pulmonary vascular (including pulmonary arteries and pulmonary veins), trachea, and bronchi, was evaluated under the pulmonary window, and the image noise and artifacts were evaluated under the mediastinal window initially. The readers were allowed to adjust the image display window settings while evaluating the cases.

**TABLE 1:** Criteria for image quality evaluation

Score	Noise	Artifacts	Visualization of small structures and lesions
5	Minimal image noise	No artifacts	Excellent visualization with very clear margins
4	Less than average noise	Minor artifacts not interfering with diagnostic decision making	Good visualization with slightly blurred margins
3	Average image noise	Some artifacts affecting visualization of small structures, diagnosis still possible	Acceptable visualization with some blurred margins
2	Above average noise	Major artifacts affecting visualization of major structures, diagnosis not certain	Uncertain in visualization with blurred margins
1	Unacceptable image noise	Severe artifacts affecting diagnosis	Unable to define object and margins

### 2.4 Statistical analysis

SPSS26.0 was used for statistical analysis. The objective image quality (CT attenuation value and SDs) was compared using repeated‐measures ANOVA with Bonferroni correction, and Friedman's test was used to compare the subjective image quality scores. A *p* value <0.05 was considered statistically significant. Kappa statistics was used to test the consistency of subjective image quality analysis between the two readers. A Kappa value between 0.81 and 1.00 represents excellent consistency; 0.61–0.80 represents substantial consistency; 0.41–0.60 represents moderate consistency; 0.21–0.40 represents fair consistency; 0.00–0.20 represents poor consistency.

## 3 RESULTS

### 3.1 Patient population

The study group consisted of 86 patients (80 men and 6 female) with a mean age of 64 ± 9 years (range, 49–86 years), a mean weight of 74.5 ± 6.1 kg (range, 44–100 kg), and a mean body mass index of 25.38 ± 4.85 kg/m^2^ (range, 16.54–35.43). The effective radiation dose for the patient group was 1.03 ± 0.36 mSv.

### 3.2 Objective image quality

The CT attenuation values and SDs of ASiR‐V40%, DLIR‐L, DLIR‐M, and DLIR‐H are shown in Table 2. There was no significant difference in CT attenuation values of the descending aorta and erector spinal muscle among the four reconstruction groups. Although there were differences in the CT attenuation values of lung tissue and fat in the four different reconstruction groups, the differences in CT attenuation values between every two groups were less than 5 HU. The image noise in descending aorta, fat, erector spinal muscle, and lung tissue showed statistically significant differences between every two different reconstruction groups (all *p* < 0.01), and DLIR‐H had the lowest noise value. Compared with ASiR‐V40%, the image noise of DLIR‐L, DLIR‐M, and DLIR‐H was reduced by 19.18%, 28.28%, and 33.56% in lung tissue, 9.28%, 29.63%, and 51.13% in erector spinae muscles, 14.07%, 35.53%, and 58.31% in fat, and 11.44%, 33.03%, and 56.1% in descending aorta, respectively.

**TABLE 2:** Comparison of objective image quality among four reconstruction groups

							*P*2					
Parameters	Tissue	ASiR‐V40%	DLIR‐L	DLIR‐M	DLIR‐H	*P*1	ASiR‐V40% versus DLIR‐L	ASiR‐V40% versus DLIR‐M	ASiR‐V40% versus DLIR‐H	DLIR‐L versus DLIR‐M	DLIR‐L versus DLIR‐H	DLIR‐M versus DLIR‐H
CT attenuation value (HU)	Lung	−896.12 ± 25.41	−895.25 ± 24.94	−894.72 ± 24.87	−893.73 ± 24.96	<0.001	0.002	<0.001	<0.001	<0.001	<0.001	<0.001
	Muscle	51.71 ± 9.93	51.57 ± 8.31	51.48 ± 7.57	51.39 ± 6.87	0.53	1.00	1.00	1.00	1.00	1.00	1.00
	Fat	−110.30 ± 7.93	−109.21 ± 6.71	−108.77 ± 6.33	−108.45 ± 5.89	<0.001	<0.001	<0.001	<0.001	0.002	0.001	0.053
	Aorta	49.12 ± 6.73	49.37 ± 5.82	49.63 ± 5.25	49.24 ± 4.64	0.23	0.97	0.67	1.00	1.00	1.00	0.30
Noise (HU)	Lung	20.86 ± 4.43	16.86 ± 3.74	14.96 ± 3.87	13.86 ± 3.25	<0.001	<0.001	<0.001	<0.001	<0.001	<0.001	<0.001
	Muscle	21.77 ± 3.16	19.75 ± 2.73	15.32 ± 2.23	10.64 ± 1.84	<0.001	<0.001	<0.001	<0.001	<0.001	<0.001	<0.001
	Fat	17.20 ± 3.54	14.78 ± 2.99	11.09 ± 2.42	7.17 ± 1.91	<0.001	<0.001	<0.001	<0.001	<0.001	<0.001	<0.001
	Aorta	21.16 ± 3.73	18.74 ± 3.01	14.17 ± 2.28	9.29 ± 1.46	<0.001	<0.001	<0.001	<0.001	<0.001	<0.001	<0.001

*Note*: Data given is mean ± SD. *P*1 means the *p* value among whole four groups (ASiR‐V40%, DLIR‐L, DLIR‐M, and DLIR‐H groups), *P*2 means the *p* value between every two groups.

Abbreviations: ASiR‐V, adaptive statistical iterative reconstruction‐Veo; DLIR‐H, deep‐learning image reconstruction at high level; DLIR‐L; deep learning image reconstruction at low level; DLIR‐M, deep learning image reconstruction at medium level; HU, Hounsfield units; SD, standard deviation.

### 3.3 Subjective image quality

**FIGURE 1:**
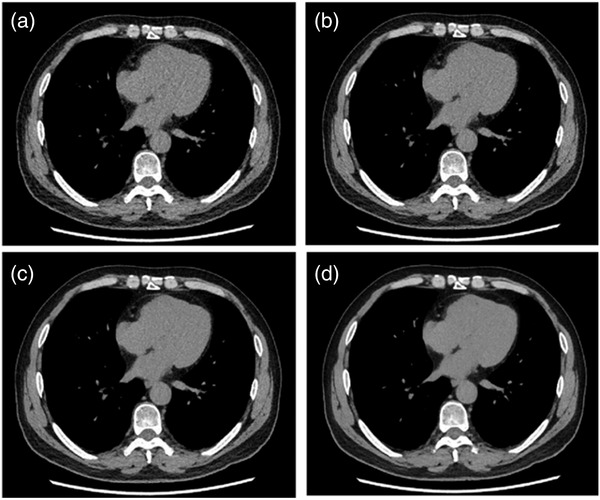
A 66‐year‐old male. (a) Axial CT image reconstructed with adaptive statistical iterative reconstruction V 40%. Axial CT images reconstructed with deep learning image reconstruction (DLIR) at low‐strength (b), DLIR at medium strength (c), and DLIR at high‐strength (d). Deep‐learning image reconstruction at high level (DLIR‐H) image had the lowest image noise and highest overall image quality under the mediastinal window display.

The results of subjective image analyses are shown in Table 3 (average scores) and Table 4 (score distribution). DLIR‐L (3.08), DLIR‐M (3.92), and DLIR‐H (4.94) had higher subjective scores in image noise than ASiR‐V40% (2.09) (Figure 1). There were significant differences among the four reconstruction groups and between each of the two groups (all *p* < 0.001). In terms of image artifacts, there were significant differences between ASiR‐V40% and DLIR reconstruction groups (ASiR‐V40% vs. DLIR‐L, ASiR‐V40% vs. DLIR‐M, and ASiR‐V40% vs. DLIR‐H, all *p* < 0.001), but the three DLIR groups had similar results (DLIR‐L vs. DLIR‐M, DLIR‐L vs. DLIR‐H, and DLIR‐M vs. DLIR‐H, all *p* = 1.00). In the visualization of small structures, ASiR‐V40%, DLIR‐L, DLIR‐M, and DLIR‐H scored 4.88 ± 0.32, 4.78 ± 0.47, 4.76 ± 0.53, 4.13 ± 0.37, respectively. Compared with DLIR‐H, the ASiR‐V40%, DLIR‐L, and DLIR‐M images were ranked higher in the qualitative score for the visualization of small structures (all *p* < 0.001), whereas ASiR‐V40%, DLIR‐L, and DLIR‐M had equivalent score (ASiR‐V40% vs. DLIR‐L, ASiR‐V40% vs. DLIR‐M, and DLIR‐L vs. DLIR‐M, *p* = 0.71, 0.47, and 1.00, respectively). There were excellent interobserver agreements in the subjective image quality evaluation between the two readers (Kappa values ranged 0.883–0.927).

**TABLE 3:** Comparison of subjective image quality among four reconstruction groups

						*P*2					
Image assessment terms	ASiR‐V40%	DLIR‐L	DLIR‐M	DLIR‐H	*P*1	ASiR‐V40% versus DLIR‐L	ASiR‐V40% versus DLIR‐M	ASiR‐V40% versus DLIR‐H	DLIR‐L versus DLIR‐M	DLIR‐L versus DLIR‐H	DLIR‐M versus DLIR‐H
Image noise	2.09 ± 0.29	3.08 ± 0.35	3.92 ± 0.28	4.94 ± 0.24	<0.001	<0.001	<0.001	<0.001	<0.001	<0.001	<0.001
Image artifacts	4.31 ± 0.49	4.90 ± 0.31	4.94 ± 0.24	4.93 ± 0.26	<0.001	<0.001	<0.001	<0.001	1.00	1.00	1.00
Visualization of small structures and lesions	4.88 ± 0.32	4.78 ± 0.47	4.76 ± 0.53	4.13 ± 0.37	<0.001	0.71	0.47	<0.001	1.00	<0.001	<0.001

*Note*: Data given is mean ± SD. *P*1 means the *p* value among whole four groups (ASiR‐V40%, DLIR‐L, DLIR‐M, and DLIR‐H groups), *P*2 means the *p* value between every two groups.

Abbreviations: ASiR‐V, adaptive statistical iterative reconstruction‐Veo; DLIR‐H, deep‐learning image reconstruction at high level; DLIR‐L, deep learning image reconstruction at low level; DLIR‐M, deep‐learning image reconstruction at medium level.

**TABLE 4:** The distribution of subjective scores among four reconstruction groups

Reconstruction		ASiR‐V40%					DLIR‐L					DLIR‐M					DLIR‐H				
**Score**		1	2	3	4	5	1	2	3	4	5	1	2	3	4	5	1	2	3	4	5
**Reader 1**	**Image noise**	0	78	8	0	0	0	2	75	9	0	0	0	7	79	0	0	0	0	5	81
	**Image artifacts**	0	0	1	57	28	0	0	0	9	77	0	0	0	5	81	0	0	0	6	80
	**Visualization of small structures and lesions**	0	0	0	10	76	0	0	2	15	69	0	0	4	13	69	0	0	1	73	12
**Reader 2**	**Image noise**	0	79	7	0	0	0	2	75	9	0	0	0	8	78	0	0	0	0	4	82
	**Image artifacts**	0	0	2	54	30	0	0	0	7	79	0	0	0	6	80	0	0	0	5	81
	**Visualization of small structures and lesions**	0	0	0	10	76	0	0	4	13	69	0	0	2	14	70	0	0	0	75	11

Abbreviations: DLIR‐H, deep learning image reconstruction at high level; DLIR‐L, deep learning image reconstruction at low level; DLIR‐M, deep learning image reconstruction at medium level.

## 4 DISCUSSION

In this study, we compared the image quality of DLIR and ASiR‐V40% in LDCT scan of the chest. Our results showed that DLIR significantly reduced image noise and artifacts and improved the image quality at descending aorta, fat, erector spinae muscles, and lung tissue, while maintaining similar ability to visualize small structures with DLIR‐L and DLIR‐M algorithms.

DLIR reconstructions brought favorable results supported by the quantitative measurements and subjective image quality scores. DLIR‐H performed best in terms of image noise, producing noise in fat that was 58.31% lower than ASiR‐V40%, whereas DLIR‐M had the best performance in reducing image artifacts. ASiR‐V40%, DLIR‐L, and DLIR‐M provided similar high scores in the visualization of small structures, whereas DLIR‐H appeared slightly over‐smoothed. Readers indicated that the reconstructed images of ASiR‐V40% and DLIR‐L had granular artifacts due to higher image noise. Achieving lower noise has historically been associated with higher radiation doses or undesirable image noise texture with conventional denoising algorithms.^10^ Surprisingly, the typical tradeoff between noise reduction and undesirable texture with high‐level IR algorithms was not pronounced with DLIR in our study, suggesting that DLIR could reduce image noise, while maintaining image texture. However, small structures, such as bronchioles, might be challenging to visualize with the highest DLIR strength. DLIR‐M may be a better choice for this purpose.

**FIGURE 2:**
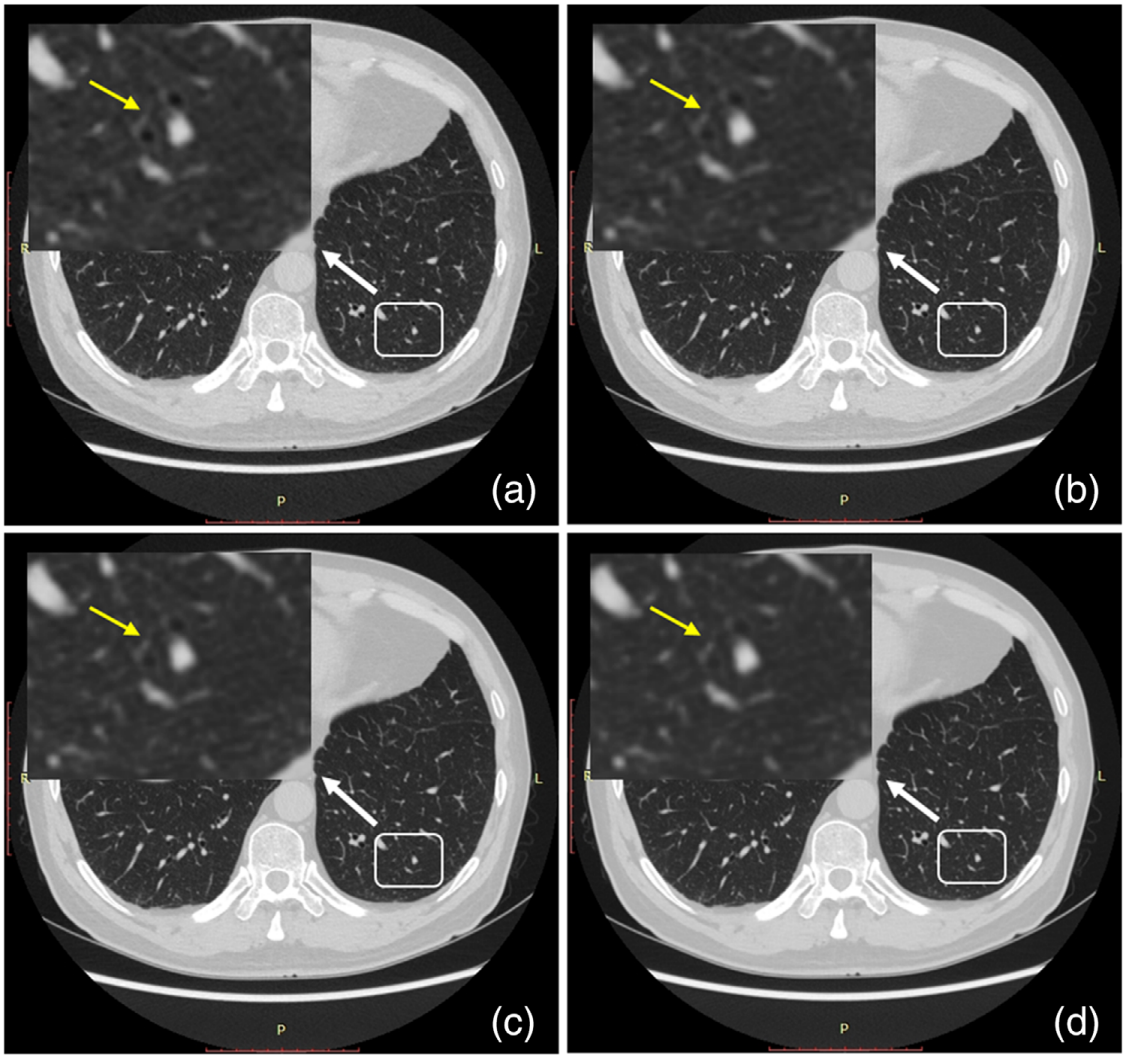
A 66‐year‐old male. (a) Axial CT image reconstructed with adaptive statistical iterative reconstruction V 40%. Axial CT images reconstructed with deep learning image reconstruction (DLIR) at low‐strength (b), DLIR at medium strength (c), and DLIR at high‐strength (d). Deep‐learning image reconstruction at medium level (DLIR‐M) best balanced image noise and the display of the small bronchi indicated by the arrows, whereas deep‐learning image reconstruction at high level (DLIR‐H) had slight blurring.

Since the introduction of DLIR, clinical studies based on DLIR have reported significant noise reduction in coronary CT angiography,^11^, ^12^, ^13^ abdominal CT scan,^14^, ^15^, ^16^, ^17^ and low‐dose chest CT scan,^18^ resulting in superior image quality with satisfactory image texture. Our results also strongly supported the fact that DLIR could significantly reduce image noise in low‐dose chest CT scans, similar to the study of Kim et al.^18^ In addition, we also tried to answer the following question: Does DLIR lose image detail as the strength of the algorithm increases as commonly seen in conventional IR algorithms?^19^, ^20^ We found that with DLIR‐M, the display and clarity of small branches of the pulmonary vascular and the small branches of the bronchi under the lung window were not negatively impacted, even though image noise was still significantly reduced. However, DLIR‐H images showed slight blurring at the edges of the previous structures (Figure 2). This result was similar to that of Jensen et al.^16^ who pointed out minor blurring of very small lesions (<5 mm) and vessels when using DLIR‐H. As the exams in our study were performed using the standard dose employed in our routine clinical protocol, it is not clear from our results how DLIR‐H would perform in lower‐dose applications. As the DLIR‐M reconstruction effectively balanced both the image noise and the display of small structures, we would recommend the use of DLIR‐M to reconstruct images in daily work at routine radiation dose levels. On the other hand, with greater power of DLIR‐H in image noise reduction, we would recommend the use of DLIR‐H when low density resolution is the focus or further radiation dose reduction is in demand.

The current study had some limitations. First, these patients were under routine physical examination for lung cancer screening, and no space‐occupying lesions were found, task‐specific lesion detection, and characterization studies are necessary to further determine the diagnostic performance of DLIR. Second, this was a retrospective study, so it could not reflect the dose reduction ability of DLIR, but through this study we had confirmed that DLIR could significantly reduce image noise and improve image quality, and the noise reduction ability may be converted in the future to radiation dose reduction. However, such dose reduction ability certainly needs further confirmation. Third, the special feature of DLIR is its ability to improve the image quality while maintaining the spatial resolution of the image. Readers in our study also had this impression, but we did not evaluate the spatial resolution of the image in detail, and we will investigate this issue in the next step. Fourth, as ASIR‐V40% is the standard reconstruction algorithm and setting for the routine clinical use in our hospital, we did not compare DLIR with ASIR‐V of other strengths. Moreover, we did not compare DLIR with other reconstruction algorithms such as FBP (which could be considered as ASIR‐V of 0%) and the full model‐based IR, which is not available in our country. Further studies involving different IR levels and different reconstruction algorithms are needed, especially when evaluating lesion detection and diagnosis.

In conclusion, compared with ASiR‐V40%, DLIR can effectively reduce image noise and significantly improve image quality in low‐dose chest CT. It is feasible to select the appropriate reconstruction intensity of DLIR in clinical practice that provides the best overall balanced image quality according to clinical tasks and DLIR‐M is better suited in low‐dose chest CT.

## AUTHOR CONTRIBUTIONS

Guarantor of integrity of the entire study: Jianxin Guo, Qian Tian, Xinyu Li. Study concepts and design: Jianxin Guo, Qian Tian, Xinyu Li, Jianying Li. Literature research: Qian Tian, Xinyu Li, Jianying Li. Data analysis: Qian Tian, Xinyu Li, Yannan Cheng, Xinyi Niu, Shumeng Zhu. Statistical analysis: Qian Tian, Xinyu Li, Shumeng Zhu, Wenting Xu. Manuscript preparation: Qian Tian, Xinyu Li, Yannan Cheng, Xinyi Niu. Manuscript editing: Qian Tian, Xinyu Li, Jianying Li.

## CONFLICT OF INTEREST

None.

## Data Availability

The data that support the findings of this study are available on request from the corresponding author. The data are not publicly available due to privacy or ethical restrictions.
